# A New Power Topp–Leone distribution with applications to engineering and industry data

**DOI:** 10.1371/journal.pone.0278225

**Published:** 2023-01-17

**Authors:** Mintodê Nicodème Atchadé, Melchior N’bouké, Aliou Moussa Djibril, Shabnam Shahzadi, Eslam Hussam, Ramy Aldallal, Huda M. Alshanbari, Ahmed M. Gemeay, Abdal-Aziz H. El-Bagoury

**Affiliations:** 1 National Higher School of Mathematics Genius and Modelization, National University of Sciences, Technologies, Engineering and Mathematics, Abomey, Benin Republic; 2 University of Abomey-Calavi/International Chair in Mathematical Physics and Applications (ICMPA : UNESCO-Chair), Cotonou, Rep. Benin; 3 Department of Statistics and Econometrics, Saint-Petersburg State University of Economics, Saint-Petersburg, Russian Federation; 4 Department of Mathematics and Big Data, Anhui University of Science and Technology, Huainan, China; 5 Department of Mathmematics, Faculty of Science, Helwan University, Cairo, Egypt; 6 Department of Accounting, College of Business Administration in Hawtat Bani Tamim, Prince Sattam Abdulaziz University, Al-Kharj, Saudi Arabia; 7 Department of Mathematical Sciences, College of Science, Princess Nourah Bint Abdulrahman University, Riyadh, Saudi Arabia; 8 Department of Mathematics, Faculty of Science, Tanta University, Tanta, Egypt; 9 Basic Science Department, Higher Institute of Engineering and Technology, El-Mahala El-Kobra, Egypt; Amity University - Lucknow Campus, INDIA

## Abstract

We introduced a brand-new member of the family that is going to be referred to as the New Power Topp-Leone Generated (NPTL-G). This new member is one of a kind. Given the major functions that created this new member, important mathematical aspects are discussed in as much detail as possible. We derived some functions for the new one, included the Rényi entropy, the qf, series development, and moment weighted probabilities. Moreover, to estimate the values of the parameters of our model that were not known, we employed the maximum likelihood technique. In addition, two actual datasets from the real world were investigated in order to bring attention to the possible applications of this novel distribution. This new model performs better than three key rivals based on the measurements that were collected.

## 1. Introduction

The development of efficient and flexible statistical models is gaining momentum due to the ever-increasing amount of data from various application domains and the inexorable advances in computer science. These models can be obtained from a broad class of distributions with the appropriate characteristics, such those created by generated distributions. Our approache’s major goal is to provide a more adaptable new cumulative density function (CDF) that depends on the inverse Lomax model. By modifying the inverse exponential distribution, we proposed a new modification of the inverse exponential distribution. This modification is a novelty in the literature. The following are some prominent instances of such families: Poisson-G [1], Odd Fréchet-G [2], Truncated inverse Kumaraswamy-G [3], New Power of Topp-Leone-G [4], Introduction to the generalized Topp-Leone family [5], Garhy-G [6], Inverse-Lomax power [7], Half-Logistic-G type II [8], Topp-Leone Inverse Lomax [9], Topp-Leone-Weibull [10], temporal distribution [11], Topp-Leone distribution, estimation [12], Topp-Leone family of distributions and some of its application on real data and some of its statsistical properties [13], moments of order statistics of Topp-Leone distribution [14] Fréchet Topp-Leone-G [15], Topp-Leone G transmuted [16], new insights on goodness-of-fit tests [17], a generalized Birnbaum-Saunders distribution [18], efficient reliability estimation in two-parameter exponential distributions [19], the Marshall-Olkin extended generalized Rayleigh distribution [20], tests to determine whether or not the Rayleigh distribution is a good fit [21], Bayesian analysis [22, 23] is also of big interest in our study.

Let’s take a closer look at the Topp-Leone-G power family so that we may accomplish the purpose of this work.

The CDF of the Topp-Leone distribution is given by:

$$\begin{array}{c} F(x;\alpha )=x^{\alpha }{\left(2-x\right)}^{\alpha }, \end{array}$$ (1)

where *x* ∈ (0, 1) and *α* > 0.

The family of distributions known as Topp-Leone-G is constructed by combining *F* by *K*, where *K* represents a CDF. The particular CDF represents this family:

$$\begin{array}{c} F(x;\alpha ,\beta ,\sigma )={\left[K(x;\sigma )\right]}^{\alpha \beta }{\left(2-{\left[K(x;\sigma )\right]}^{\beta }\right)}^{\alpha },x\in R. \end{array}$$ (2)

*K* (*x*; *σ*) ∈ [0, 1], *α*, *β* > 0 and *K*(*x*;*σ*) is the CDF of a basic continuous distribution dependent on *σ* = (*σ*_1_, …, *σ*_*n*_).

Through the use of the CDF function of the inverse exponential distribution and *K*(*x*;*σ*), so we can easily obtain:

$$\begin{array}{c} F(x;\sigma )=e^{1-\frac{1}{K(x;\sigma )}}. \end{array}$$ (3)

These families are very simple, do not imply more parameters and present some properties that are different from the distribution *K*(*x*, *σ*). In [4], the families of Power Topp-Leone Generated (PTL-G) and Inverse Exponential Generated (IE-G) were combined by the authors to create a new family of distributions called the Inverse Exponential Generated Family. The associated CDF may be obtained from:

$$\begin{array}{c} F(x;\eta )=e^{\alpha \beta (1-\frac{1}{K(x;\sigma )})}{\left(2-e^{\beta (1-\frac{1}{K(x;\sigma )})}\right)}^{\alpha }, \end{array}$$ (4)

*η* = (*α*, *β*, *σ*);*α*, *β*, *σ* > 0. This CDF is a modification of the standard CDF that uses polyno-exponential functions *K*(*x*;*σ*). According to [4], the motivations of the NPTL-G family are to increase the flexibility of current distributions on different labels to offer better fits than rival models.

In light of the theory discussed above, we suggest a specific NPTL-G family member with the CDF *K*(*x*;*σ*) as the basic distribution. The distribution that results is the Modified Topp-Leone Inverse Lomax power distribution (MPTLILx) which has three parameters and potential applications. To illustrate this point, we will be using two different sets of real-world data. The first one is an engineering data and the second one is about the average thickness of electronic devices. The proposed model outperformed it’s competitors in terms of results, which encourages its usage for more general statistical goals.

## 2. A special member: The MPTLILx distribution

There are various distributions in the NPTL-G family. A new distribution can be discovered similarly to a new base distribution. In this study, the fundamental distribution used to define the MPTLILx distribution is the inverse of the Lomax inverse exponential with the shape parameter *θ* greater than zero. As a result, the following CDF describes it:

$$\begin{array}{c} K(x;\theta )=\frac{1}{\psi },\;\;x>0, \end{array}$$ (5)

where

$$\begin{array}{c} \psi =e^{\frac{1}{R(x;\theta )}-1}, \end{array}$$ (6)

$$\begin{array}{c} R(x;\theta )=C^{-\theta }, \end{array}$$ (7)

$$\begin{array}{c} C=1+x^{-1}. \end{array}$$ (8)

The related probability density function (pdf) is given by:

$$\begin{array}{c} k(x;\theta )=\frac{\theta C^{\theta -1}}{x^{2}\psi },\;\;x>0, \end{array}$$ (9)

and the hazard rate function (hrf) given by:

$$\begin{array}{c} h(x;\theta )=\frac{\theta C^{\theta -1}}{x^{2}\psi \left[1-\psi^{-1}\right]}. \end{array}$$ (10)

In point of fact, the MPTLILx distribution is characterized by the CDF as follows:

$$\begin{array}{c} F(x;\Theta )=e^{\alpha \beta \Psi }{\left\{2-e^{\beta \Psi }\right\}}^{\alpha }, \end{array}$$ (11)

where Θ = (*α*, *β*, *θ*), Ψ = 1 − *ψ*, and *θ* > 0, where *θ* is vector of the parameters used in this study. The following expressions will provide the pdf and hrf values that correspond to (11)

$$\begin{array}{c} f(x;\Theta )=2\alpha \beta \theta x^{-2}C^{\theta -1}e^{\left[(C^{\theta }-1)+\alpha \beta \Psi \right]}\{1-e^{\beta \Psi }\}{\left\{2-e^{\beta \Psi }\right\}}^{\alpha -1}, \end{array}$$ (12)

and

$$\begin{array}{c} h(x;\Theta )=\frac{f(x;\Theta )}{1-e^{\alpha \beta \Psi }{\left\{2-e^{\beta \Psi }\right\}}^{\alpha }}, \end{array}$$ (13)

**Fig 1:**
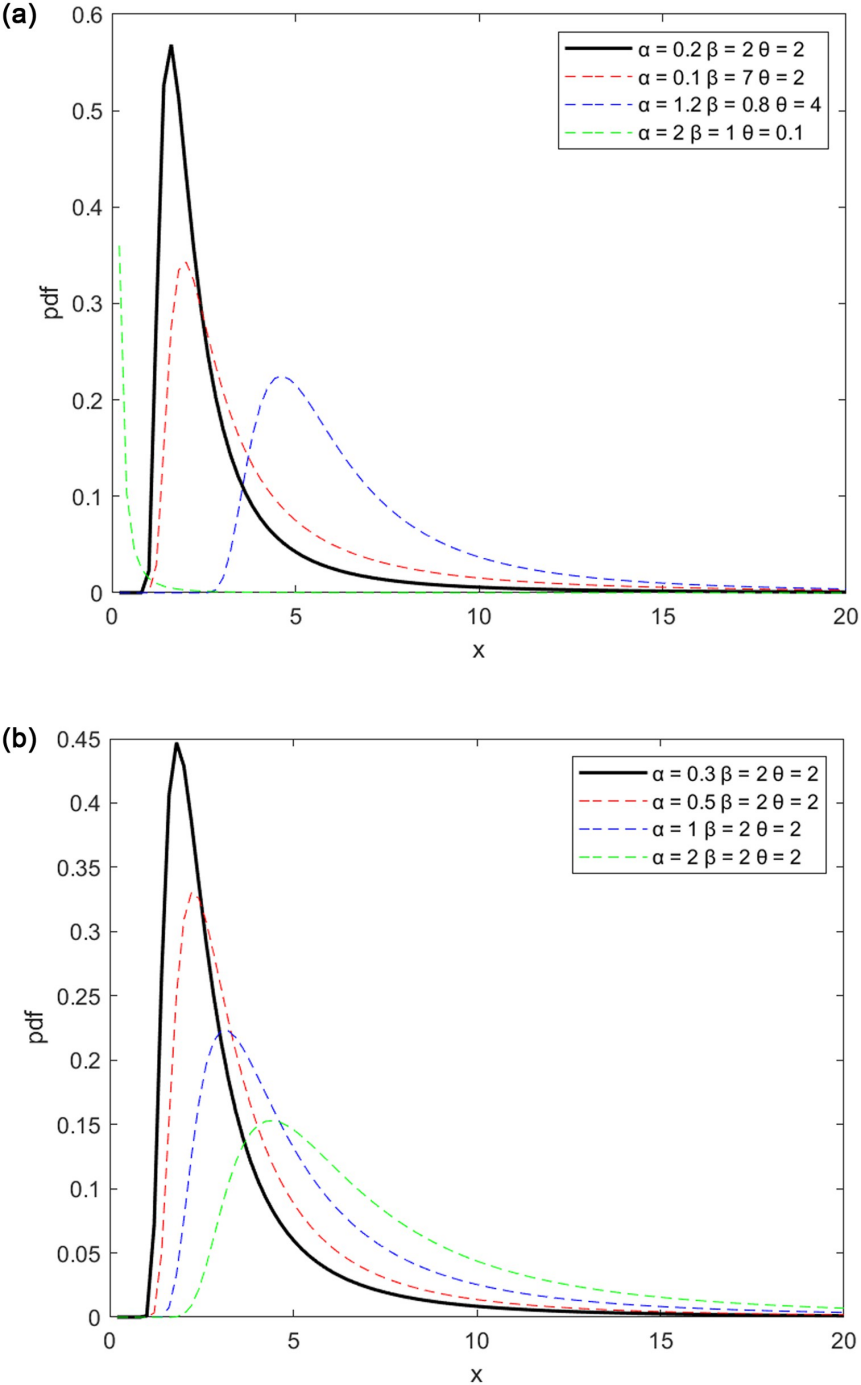
Probability density functions of the new MPTLILx. (a) For different parameter sets. (b) For different values of *α* and *β* = 2 and *θ* = 2.

**Fig 2:**
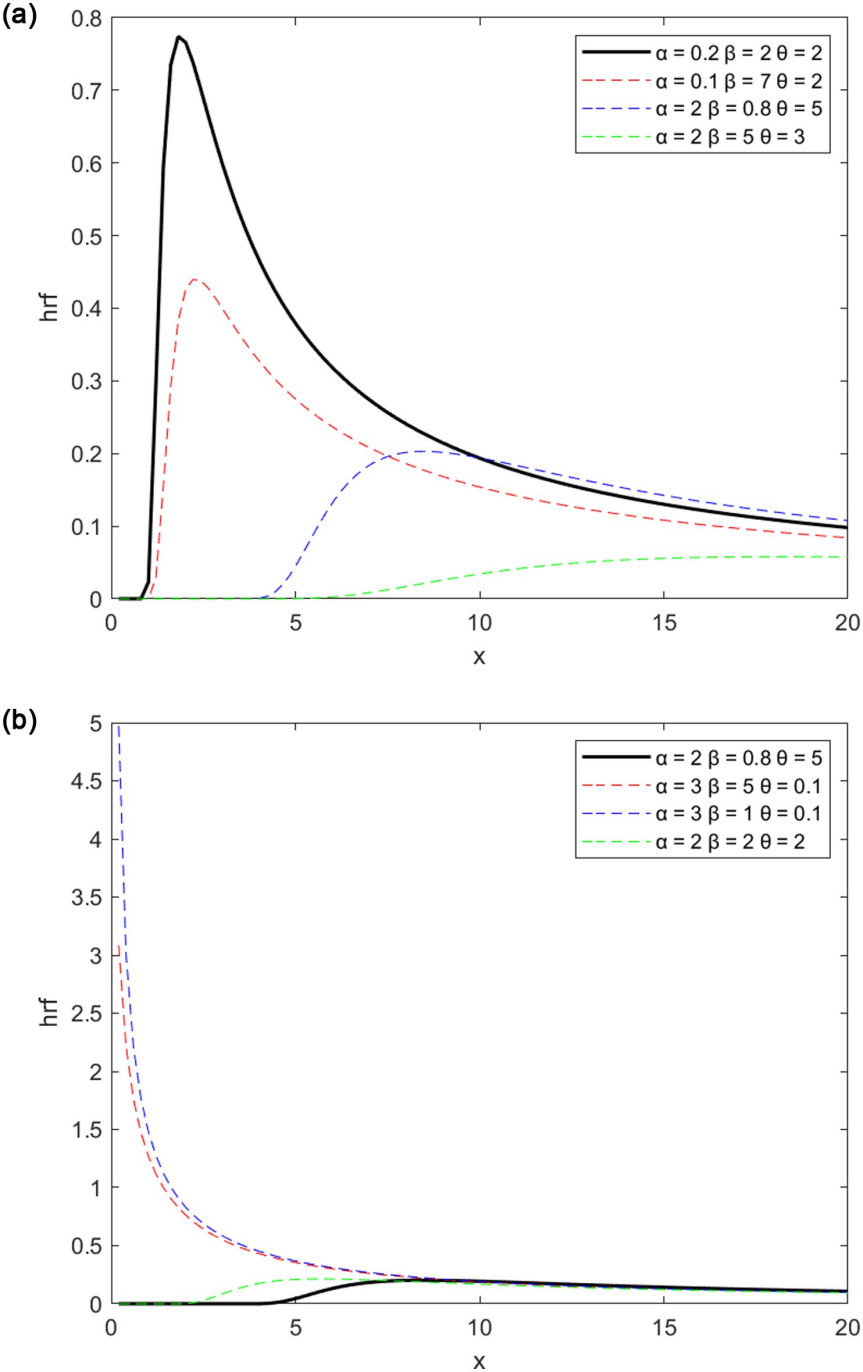
New MPTLILx’s hazard rate functions. (a) For different parameter sets. (b) For another additional group of parameters.

Figs 1 and 2, respectively depict the potential forms of the MPTLILx distribution’s pdf and hrf. Fig 1 in particular demonstrates how the pdf can have a right skew and an inverted J shape. The pdf can be growing, decreasing, inverted, or bathtub-shaped as in [4], as seen in Fig 2. It is well recognized that all of these curvature characteristics are ideal for developing adaptable statistical models.

## 3. Some MPTLILx distribution mathematical properties

Some significant MPTLILx distribution mathematical features are presented in this section.

### 3.1 Rényi Entropy

**Proposition 1**

The Rényi entropy of the MPTLILx distribution for *γ* ≠ 1 is provided as below:

$$\begin{array}{c} \nu_{R}\left(X\right)=\frac{1}{1-\gamma }log\{\sum_{i=0}^{\gamma (\alpha -1)}\sum_{j=0}^{\gamma }\sum_{k=0}^{\infty }\sum_{l=0}^{k}\sum_{m=0}^{\infty }L_{i,j,k,l,m}I_{m}(\gamma ,\theta )\}, \end{array}$$ (14)

where

$$\begin{array}{c} \begin{array}{cc} L_{i,j,k,l,m} & =\frac{{\left(-1\right)}^{i+j+l}}{i!j!k!l!m!}{\left(2\alpha \beta \theta \right)}^{\gamma }2^{\gamma \left(\alpha -1\right)-i}\frac{\Gamma \left(k+1\right)\Gamma \left(\gamma +1\right)\Gamma \left(\gamma \left(\alpha -1\right)+1\right)}{\Gamma \left(k-l+1\right)\Gamma \left(\gamma -j+1\right)\Gamma \left(\gamma \left(\alpha -1\right)+1-i\right)}\\ & \times {\left(\gamma \alpha \beta +\beta \left(i+j\right)\right)}^{k}{\left(\gamma +l\right)}^{m}, \end{array} \end{array}$$ (15)

$$\begin{array}{c} I_{m}\left(\gamma ,\theta \right)=\int_{R}x^{-2\gamma }C^{\gamma \left(\theta -1\right)}{\left[C^{\theta }-1\right]}^{m}dx. \end{array}$$ (16)

**Proof**.

The Rényi entropy of *X* for continuous random variable with range *R* reflects the degree of uncertainty. One possible formulation of the definition is as follows:

$$\begin{array}{c} \nu_{R}\left(X\right)=\frac{1}{1-\gamma }log\{\int_{R}f{\left(x;\eta \right)}^{\gamma }dx\},\gamma >0,\gamma \neq 1. \end{array}$$ (17)

Let’s provide an explicit expression for *f*(*x*, *η*)^*γ*^ to obtain the Rényi entropy of the MPTLILx distribution:

$$\begin{array}{c} f(x;\eta )=2\alpha \beta \theta x^{-2}C^{\theta -1}e^{\left[(C^{\theta }-1)+\alpha \beta \Psi \right]}\{1-e^{\beta \Psi }\}{\left\{2-e^{\beta \Psi }\right\}}^{\alpha -1}, \end{array}$$ (18)

so,

$$\begin{array}{c} f{\left(x;\Theta \right)}^{\gamma }={\left(2\alpha \beta \theta \right)}^{\gamma }x^{-2\gamma }C^{\gamma \left(\theta -1\right)}e^{\gamma \left[\left(C^{\theta }-1\right)+\alpha \beta \Psi \right]}{\left\{1-e^{\beta \Psi }\right\}}^{\gamma }{\left\{2-e^{\beta \Psi }\right\}}^{\gamma \left(\alpha -1\right)}, \end{array}$$ (19)

$$\begin{array}{cc} f{\left(x;\Theta \right)}^{\gamma } & =\sum_{i=0}^{\gamma \left(\alpha -1\right)}\sum_{j=0}^{\gamma }\sum_{k=0}^{\infty }\sum_{l=0}^{k}\sum_{m=0}^{\infty }\frac{{\left(-1\right)}^{i+j+l}}{i!j!k!l!m!}{\left(2\alpha \beta \theta \right)}^{\gamma }2^{\gamma \left(\alpha -1\right)-i}\frac{\Gamma \left(k+1\right)\Gamma \left(\gamma +1\right)\Gamma \left(\gamma \left(\alpha -1\right)+1\right)}{\Gamma \left(k-l+1\right)\Gamma \left(\gamma -j+1\right)\Gamma \left(\gamma \left(\alpha -1\right)+1-i\right)}\\ & \times {\left(\gamma \alpha \beta +\beta \left(i+j\right)\right)}^{k}{\left(\gamma +l\right)}^{m}x^{-2\gamma }C^{\gamma \left(\theta -1\right)}{\left[C^{\theta }-1\right]}^{m}. \end{array}$$ (20)

As a result, the Renyi entropy of the MPTLILx distribution is provided for *γ* ≠ 1 by:

$$\begin{array}{c} \nu_{R}\left(X\right)=\frac{1}{1-\gamma }log\{\sum_{i=0}^{\gamma \left(\alpha -1\right)}\sum_{j=0}^{\gamma }\sum_{k=0}^{\infty }\sum_{l=0}^{k}\sum_{m=0}^{\infty }L_{i,j,k,l,m}I_{m}\left(\gamma ,\theta \right)\}, \end{array}$$ (21)

where *L*_*i*,*j*,*k*,*l*,*m*_ and *I*_*m*_(*γ*, *θ*) are provided by (15) and (16).

### 3.2 Quantile function (qf)

The following result is an expression of the qf of the MPTLILx distribution.

**Proposition 2**

The following equation describes the qf of the MPTLILx distribution:

$$\begin{array}{c} Q\left(u;\Theta \right)={\left[{\left[1+\;\text{ln}\;\left(1-\frac{1}{\beta }\;\text{ln}\;\left(1-\sqrt{1-u^{\frac{1}{\alpha }}}\right)\right)\right]}^{\frac{1}{\theta }}-1\right]}^{-1}, \end{array}$$ (22)

*u* ∈ ]0, 1[.

**Proof**.

In fact, let us pose *x*_*u*_ = *Q*(*u*; Θ) ∀*u* ∈ ]0, 1[. The qf may be understood by referring to its definition, *x*_*u*_ meets the requirements of the nonlinear equation *u* = *F*(*x*, *η*).

$$\begin{array}{cc} u & =F\left(x;\eta \right)\\ & \Rightarrow u=e^{\alpha \beta \left(1-\frac{1}{K\left(x;\sigma \right)}\right)}{\left(2-e^{\beta \left(1-\frac{1}{K\left(x;\sigma \right)}\right)}\right)}^{\alpha }, \end{array}$$

$$\begin{array}{c} \Leftrightarrow K\left(x;\sigma \right)={\left(1-\frac{1}{\beta }\;\text{ln}\;\left(1-\sqrt{1-u^{\frac{1}{\alpha }}}\right)\right)}^{-1}, \end{array}$$

$$\begin{array}{c} K\left(x;\theta \right)=\frac{1}{\psi }, \end{array}$$

so

$$\begin{array}{c} \frac{1}{\psi }={\left(1-\frac{1}{\beta }\;\text{ln}\;\left(1-\sqrt{1-u^{\frac{1}{\alpha }}}\right)\right)}^{-1}, \end{array}$$

drawing *x* in the latter expression, we get:

$$\begin{array}{c} x={\left[{\left[1+\;\text{ln}\;\left(1-\frac{1}{\beta }\;\text{ln}\;\left(1-\sqrt{1-u^{\frac{1}{\alpha }}}\right)\right)\right]}^{\frac{1}{\theta }}-1\right]}^{-1}. \end{array}$$ (23)

Hence the qf is provided by (23). Table 1 contains the values of the first quartile and other values for some measures with various values of parameters.

**Table 1:** *Q*_1_, *M*, *Q*_3_, *S* and *K* for MPTLILx.

Θ	Q_1_	M	Q_3_	S	K
(0.5, 0.5, 0.5)	0.1190	0.1715	0.2752	0.3275	1.6128
(0.5, 1.5, 1.5)	1.4527	1.9720	2.9799	0.3199	1.5917
(1.5, 2.5, 2.0)	4.7098	6.7471	10.6895	0.3186	1.5860
(2.0, 3.0, 2.5)	7.9849	11.5417	18.4039	0.3172	1.5836
(2.5, 3.5, 3.0)	12.3182	17.9194	28.6987	0.3161	1.5817
(3.0, 4.0, 3.5)	17.8243	26.0447	41.8313	0.3152	1.5803
(3.0, 4.0, 4.0)	20.4409	29.8360	47.8781	0.3152	1.5803
(3.0, 4.0, 4.5)	23.0575	33.6273	53.9250	0.3151	1.5803
(3.0, 4.0, 5.0)	25.6743	37.4188	59.9719	0.3151	1.5803
(4.5, 5.0, 6.0)	46.8404	68.6312	110.2836	0.3131	1.5775
(7.0, 6.0, 6.5)	76.6344	112.2654	180.0926	0.3112	1.5752
(7.5, 7.0, 8.0)	113.2652	166.2729	267.1325	0.3110	1.5750
(8.0, 7.5, 8.5)	133.1652	195.5465	314.1859	0.3108	1.5747
(9.0, 8.0, 8.5)	151.2158	221.9094	356.2455	0.3104	1.5743

Table 1 shows the first quartile, median and third quartile of the MPTLILx distribution. This table shows us that these values evolve as the parameters evolve. As a result, we can get an idea of what data is appropriate for our model (the large values for example for the stock market data for finance) and also an estimate of the parameters. This same table also presents the values of Skewness and Kurtosis. These values decrease as the values of the parameters increase. As a consequence, we could say that as the value of the parameters increases, the model becomes less asymmetric and less flattened.

### 3.3 Serial development of *f*

**Proposition 3**

The serial expansion that we have is as follows:

$$\begin{array}{c} f\left(x;\Theta \right)=\sum_{k=0}^{\alpha }\sum_{l=0}^{\infty }\sum_{m=0}^{l}\frac{W_{k,l,m}k_{m}\left(x;\theta \right)}{K_{m\left(m+1\right)}\left(x;\theta \right)}, \end{array}$$

where

Wk,l,m=(-1)k+m+1ml!βl(α+k)l(αk)(lm)2α-k,K(x;θ)m=Km(x;θ),([K(x;θ)]′)m=km(x;θ),K(x;θ)=1ψ.

**Proof**.

The series development of (11) is as follows:

(2-eβ(1-1K(x;θ)))α=∑k=0α(-1)k(αk)2α-keβk(1-1K(x;θ)), (24)

so,

eαβ(1-1K(x;θ))(2-eβ(1-1K(x;θ)))α=∑k=0∞(-1)k(αk)2α-keβ(α+k)(1-1K(x;θ)).

In contrast, the exponential power series provides us with the following result:

$$\begin{array}{c} e^{\beta \left(\alpha +k\right)\left(1-\frac{1}{K\left(x;\theta \right)}\right)}=\sum_{l=0}^{\infty }\frac{1}{l!}{\left(\alpha +k\right)}^{l}\beta^{l}{\left(1-\frac{1}{K\left(x;\theta \right)}\right)}^{l}, \end{array}$$

but

(1-1K(x;θ))l=∑m=0l(-1)m(lm)1K(x;θ)m,

so,

eβ(α+k)(1-1K(x;θ))=∑l=0∞∑m=0l(-1)m(lm)1l!βl(α+k)l1K(x;θ)m,

this last expression in *F* gives:

F(x;Θ)=∑k=0α∑l=0∞∑m=0l(-1)k+m1l!βl(α+k)l(αk)(lm)×2α-k1K(x;θ)m.

By deriving the latter expression with respect to *x*, we have demonstrated that Proposition 3 is true in its entirety.

### 3.4 Moment-weighted probabilities

**Proposition 4**

The (*r* + *s*)^*th*^ moment-weighted probability of the random variable *x* of the distribution denoted *M*_*r*,*s*_ is given by:

$$\begin{array}{c} M_{r,s}=\sum_{k=0}^{u}\sum_{l=0}^{\infty }\sum_{m=0}^{l}\sum_{p=0}^{\infty }\sum_{t=0}^{p}\sum_{q=0}^{w}H_{k,l,m,p,t,q,s}I_{r,q}\left(\beta ,\theta \right), \end{array}$$

where

Hk,l,m,p,t,q,s=(-1)k+m+p+tαβθ2α(s+1)-k(m+1)p(lm)(pt)(α(s+1)-1k)(θ(t+1)-1q)1l!p!(αβ+βk+αβs)l,

$$\begin{array}{c} I_{r,q}\left(\beta ,\theta \right)=\int_{R}x^{r-\left(q+2\right)}\{1-e^{\beta \Psi }\}dx. \end{array}$$

**Proof**

Let’s put Φ(*x*;Θ) = *f*(*x*;Θ) *F*^*s*^ (*x*;Θ),

$$\begin{array}{cc} \Phi \left(x;\Theta \right) & =2\alpha \beta \theta x^{-2}C^{\theta -1}e^{\left[\left(C^{\theta }-1\right)+\alpha \beta \Psi \right]}\{1-e^{\beta \Psi }\}{\left\{2-e^{\beta \Psi }\right\}}^{\alpha -1}\\ & \times {\left\{2-e^{\beta \Psi }\right\}}^{\alpha s}e^{\alpha \beta s\Psi }, \end{array}$$

$$\begin{array}{c} \Phi \left(x;\Theta \right)=2\alpha \beta \theta x^{-2}C^{\theta -1}e^{\left(C^{\theta }-1\right)}e^{\alpha \beta \Psi }e^{\alpha \beta s\Psi }\{1-e^{\beta \Psi }\}{\left\{2-e^{\beta \Psi }\right\}}^{\alpha \left(s+1\right)-1}, \end{array}$$

we have:

{2-eβΨ}α(s+1)-1=∑k=0u(-1)k(α(s+1)-1k)2α(s+1)-k-1eβkΨ,

where *u* = *α*(*s* + 1) − 1. So,

Φ(x;Θ)=∑k=0u(-1)kαβθx-22α(s+1)-kCθ-1(α(s+1)-1k)e(Cθ-1)eαβΨeαβsΨ×eβkΨ{1-eβΨ}.

On the other hand,

$$\begin{array}{cc} e^{\alpha \beta \Psi }e^{\alpha \beta s\Psi }e^{\beta k\Psi } & =e^{\Psi \left(\alpha \beta +\beta k+\alpha \beta s\right)}\\ & =\sum_{l=0}^{\infty }\frac{1}{l!}{\left(\alpha \beta +\beta k+\alpha \beta s\right)}^{l}\Psi^{l}, \end{array}$$

so

Φ(x;Θ)=∑k=0u∑l=0∞(-1)kαβθx-22α(s+1)-kCθ-1(α(s+1)-1k)ψ1l!(αβ+βk+αβs)lΨl×{1-eβΨ},

we also have:

Ψl=∑m=0l(-1)m(lm)em(Cθ-1),

so,

Φ(x;Θ)=∑k=0u∑l=0∞∑m=0l(-1)k+mαβθx-22α(s+1)-kCθ-1e(Cθ-1)(m+1)(lm)×(α(s+1)-1k)1l!(αβ+βk+αβs)l{1-eβΨ},

but

$$\begin{array}{c} e^{\left(C^{\theta }-1\right)\left(m+1\right)}=\sum_{p=0}^{\infty }\frac{1}{p!}{\left(m+1\right)}^{p}{\left(C^{\theta }-1\right)}^{p}, \end{array}$$

e(Cθ-1)(m+1)=∑p=0∞∑t=0p(-1)(p+t)p!(pt)(m+1)pCθt.

So,

Φ(x;Θ)=∑k=0u∑l=0∞∑m=0l∑p=0∞∑t=0p(-1)k+m+p+tαβθx-22α(s+1)-kCθt+θ-1(m+1)p(lm)×(pt)(α(s+1)-1k)1l!p!(αβ+βk+αβs)l{1-eβΨ},

Cθt+θ-1=∑q=0w(θ(t+1)-1q)x-q,

where *w* = *θ*(*t* + 1) − 1.

Hence,

Φ(x;Θ)=∑k=0u∑l=0∞∑m=0l∑p=0∞∑t=0p∑q=0w(-1)k+m+p+tαβθx-22α(s+1)-k(m+1)p(lm)(pt)×(α(s+1)-1k)(θ(t+1)-1q)x-q1l!p!(αβ+βk+αβs)l{1-eβΨ}, (25)

$$\begin{array}{c} M_{r,s}=E\left(X^{r}F^{s}\left(x\right)\right)=\int_{R}x^{r}\Phi \left(x;\Theta \right)dx. \end{array}$$ (26)

By replacing (25) in (26), we end the proof of proposition 4.

## 4. Maximum Likelihood Estimation (MLE)

This section examines the MPTLILx model as represented by the CDF provided by (11). The maximum likekihood technique is employed to estimate the parameters due to its intriguing theorical and practical aspects. Using the pdf that was previously mentioned, the likelihood and log-likelihood functions may be calculated using the following formulas:

$$\begin{array}{cc} L\left(\Theta \right) & =\prod_{i=1}^{n}f\left(x_{i};\Theta \right)\\ & ={\left(2\alpha \beta \theta \right)}^{n}\prod_{i=1}^{n}x_{i}^{-2}C_{i}^{\theta -1}e^{\left[\left(C_{i}^{\theta }-1\right)+\alpha \beta \Psi_{i}\right]}\{1-e^{\beta \Psi_{i}}\}{\left\{2-e^{\beta \Psi_{i}}\right\}}^{\alpha -1}, \end{array}$$ (27)

where

$$\begin{array}{cc} & C_{i}=1+x_{i}^{-1},\\ & \psi_{i}=e^{\frac{1}{R\left(x_{i};\theta \right)}-1},\\ & R_{i}\left(x_{i};\theta \right)=C_{i}^{-\theta },\\ & \Psi_{i}=1-\psi_{i} \end{array}$$ (28)

$$\begin{array}{cc} l\left(\Theta \right) & =\;\text{ln}\;\left(L\left(\Theta \right)\right)\\ & =n\;\text{ln}\;\left(2\right)+n\;\text{ln}\;\left(\alpha \right)+n\;\text{ln}\;\left(\beta \right)+n\;\text{ln}\;\left(\theta \right)-2\sum_{i=1}^{n}\;\text{ln}\;\left(x_{i}\right)+\left(\theta -1\right)\sum_{i=1}^{n}\;\text{ln}\;\left(C_{i}\right)\\ & +\sum_{i=1}^{n}\left(C_{i}^{\theta }-1\right)+\sum_{i=1}^{n}\left[\alpha \beta \Psi_{i}\right]+\sum_{i=1}^{n}\;\text{ln}\;\{1-e^{\beta \Psi_{i}}\}+\left(\alpha -1\right)\sum_{i=1}^{n}\;\text{ln}\;\{2-e^{\beta \Psi_{i}}\}. \end{array}$$ (29)

The MLEs are respectively $\hat{\alpha },\hat{\beta }$ and $\hat{\theta }$ are described in such a way that $L\left(\hat{\alpha },\hat{\beta },\hat{\theta }\right)={\text{max}}_{\Theta \in {\left(0,+\infty \right)}^{3}}L\left(\Theta \right)$ or $l\left(\hat{\alpha },\hat{\beta },\hat{\theta }\right)={\text{argmax}}_{\Theta \in {\left(0,+\infty \right)}^{3}}l\left(\Theta \right)$. It is possible to derive the MLEs by the upcoming steps.

$$\begin{array}{c} \frac{\partial l}{\partial \alpha }=\frac{n}{\alpha }+\beta \sum_{i=1}^{n}\Psi_{i}+\sum_{i=1}^{n}\;\text{ln}\;\{2-e^{\beta \Psi_{i}}\}, \end{array}$$ (30)

$$\begin{array}{cc} \frac{\partial l}{\partial \beta } & =\frac{n}{\beta }+\alpha \sum_{i=1}^{n}\Psi_{i}-\sum_{i=1}^{n}\frac{\Psi_{i}e^{\beta \Psi_{i}}}{1-e^{\beta \Psi_{i}}}\\ & -\left(\alpha -1\right)\sum_{i=1}^{n}\frac{\Psi_{i}e^{\beta \Psi_{i}}}{2-e^{\beta \Psi_{i}}}, \end{array}$$ (31)

$$\begin{array}{cc} \frac{\partial l}{\partial \theta } & =\frac{n}{\theta }+\sum_{i=1}^{n}\;\text{ln}\;\left(C_{i}\right)+\sum_{i=1}^{n}\left(C_{i}^{\theta }\;\text{ln}\;\left(C_{i}\right)\right)-\alpha \beta \sum_{i=1}^{n}\left(C_{i}^{\theta }\;\text{ln}\;\left(C_{i}\right)\psi_{i}\right)\\ & +\sum_{i=1}^{n}\frac{\beta C_{i}^{\theta }\;\text{ln}\;\left(C_{i}\right)\psi_{i}e^{\beta \Psi_{i}}}{1-e^{\beta \Psi_{i}}}\\ & +\left(\alpha -1\right)\sum_{i=1}^{n}\frac{\beta C_{i}^{\theta }\;\text{ln}\;\left(C_{i}\right)\psi_{i}e^{\beta \Psi_{i}}}{2-e^{\beta \Psi_{i}}}, \end{array}$$ (32)

We are unable to supply closed forms for the MLEs due to the intricacy of these expressions. However, there are a number of numerical methods based on Newton-Raphson algorithms for maximizing *l* and one of these is used in this work.

## 5. Practical example using real data

We will demonstrate the adaptability of the MPTLILx distribution by analyzing two sets of data derived from actual life events. In addition, we compared the MPTLILx model to a number of other models, some of which are listed below, to determine how well it fits the data.

1. New Power Topp-Leone Inverse Lomax (NPTLILx) [4].
2. Topp-Leone Inverse Lomax (TILx) [9].
3. Inverse Lomax (ILx) [24].

These distributions have in common the inverse of Lomax as a basic distribution, which allows comparing these models. With the reversal of the Lomax distribution being the only notable exception here [24] the models considered have three parameters. Some well-known statistical metrics such as minus log-likelihood $\left(-\hat{l}\right)$, CAIC (Corrected Akaike Information Criterion) and others were used to compare these models. Let’s recall that the best model is the one with the lowest criterion. We used MATLAB and Mathematica softwares to compute all these metrics.

Dataset I: The first piece of data is from the field of civil engineering, and it records the times at which represents the hailing times. Previous consideration of it may be found in Kotz and Van Dorp [25]. The observations are: 3.20, 3.40, 3.50, 3.50, 3.60, 3.60, 3.90, 4.15, 4.30, 4.40, 4.40, 4.40, 4.54, 4.60, 4.70, 4.70, 4.73, 4.75, 4.79, 4.80, 4.80, 4.82, 4.90, 4.95, 5.10, 5.10, 5.15, 5.20, 5.20, 5.30, 5.40, 5.40, 5.40, 5.40, 5.40, 5.60, 5.60, 5.70, 5.70, 5.70, 5.80, 5.80, 5.80, 5.80, 5.90, 5.90, 5.90, 5.90, 5.90, 5.90, 5.90, 5.90, 5.90, 5.90, 6.00, 6.00, 6.00, 6.00, 6.00, 6.00, 6.00, 6.00, 6.20, 6.30, 6.40, 6.50, 6.50, 6.50, 6.60, 6.70, 6.80, 6.80, 6.90, 7.00, 7.00, 7.00, 7.10, 7.30, 7.40, 7.50, 7.90, 8.00, 8.20, 8.50, 8.60. Table 2 contains the values of the estimates for the first data.

**Table 2:** Estimates of the hailing times data using MLEs.

Model	*α*	*β*	*θ*	λ	*γ*	*ϕ*
MPTLILx	2.5510	0.2760	4.7484	-	-	-
NPTLIEx	15.8873	0.1704	5.4952	-	-	-
TILx	5.9759 × 10^7^	-	-	-	1.6647	0.0004
ILx	-	-	-	153755	0.0000	-

Dataset II: The second data set refers to [26] and is from the industrial sector and represents the average thickness of electronic devices. The set is consisted of 24 observations which are: 6.21, 6.22, 6.47, 6.69, 6.92, 6.97, 6.99, 7.02, 7.13, 7.15, 7.19, 7.22, 7.22, 7.24, 7.24, 7.28, 7.32, 7.32, 7.44, 7.47, 7.52, 7.52, 7.61, 7.67. Table 3 contains the values of the estimates for the first data.

**Table 3:** Estimates of the second data using MLEs.

Model	*α*	*β*	*θ*	λ	*γ*	*φ*
MPTLILx	0.5308	0.0485	11.7844	-	-	-
NPTLIEx	81.4711	0.0205	15.2008	-	-	-
TILx	1.1676 × 10^6^	-	-	-	6.3391	0.0010
ILx	-	-	-	37761.7	0.0001	-

Tables 2 and 3 show us the values of the parameters estimated by maximum likelihood. These values are obtained thanks to the function *Maximize* of Mathematica software.

Tables 4 and 5 show the AIC, CAIC, BIC, HQIC, AD, W* and KS obtained for the two datasets I and II respectively.

**Table 4:** The information criteria results for the hailing time data.

Models	$-\hat{l}$	AIC	CAIC	BIC	HQIC	AD	W*	KS
MPTLILx	153.7920	313.5840	313.8803	320.9120	316.5315	4.5158	0.7406	0.1448
NPTLILx	165.5980	337.1960	337.4923	344.5240	340.1435	9.1279	1.7047	0.2632
TILx	180.0770	366.1540	366.4503	373.4820	369.1015	14.1181	2.8402	0.3357
ILx	232.9130	469.8260	469.9723	474.7113	471.7910	24.8958	5.2911	0.4689

**Table 5:** The information criteria results for the thickness of electronic devices data.

Models	$-\hat{l}$	AIC	CAIC	BIC	HQIC	AD	W*	KS
MPTLILx	21.7733	49.5466	50.2133	54.6132	51.3785	2.8138	0.5308	0.2959
NPTLILx	38.8239	83.6478	84.3145	88.7144	85.4797	9.7391	2.1915	0.6007
TILx	54.6278	115.2556	115.9223	120.3222	117.0875	8.8708	1.9248	0.5748
ILx	71.1327	146.2654	146.5897	149.6432	147.4867	9.9094	2.1641	0.6039

We see that these statistics for the MPTLILx model are smaller than those of the four competing models NPTLILx, TILx and ILx for datasets I and II.

**Fig 3:**
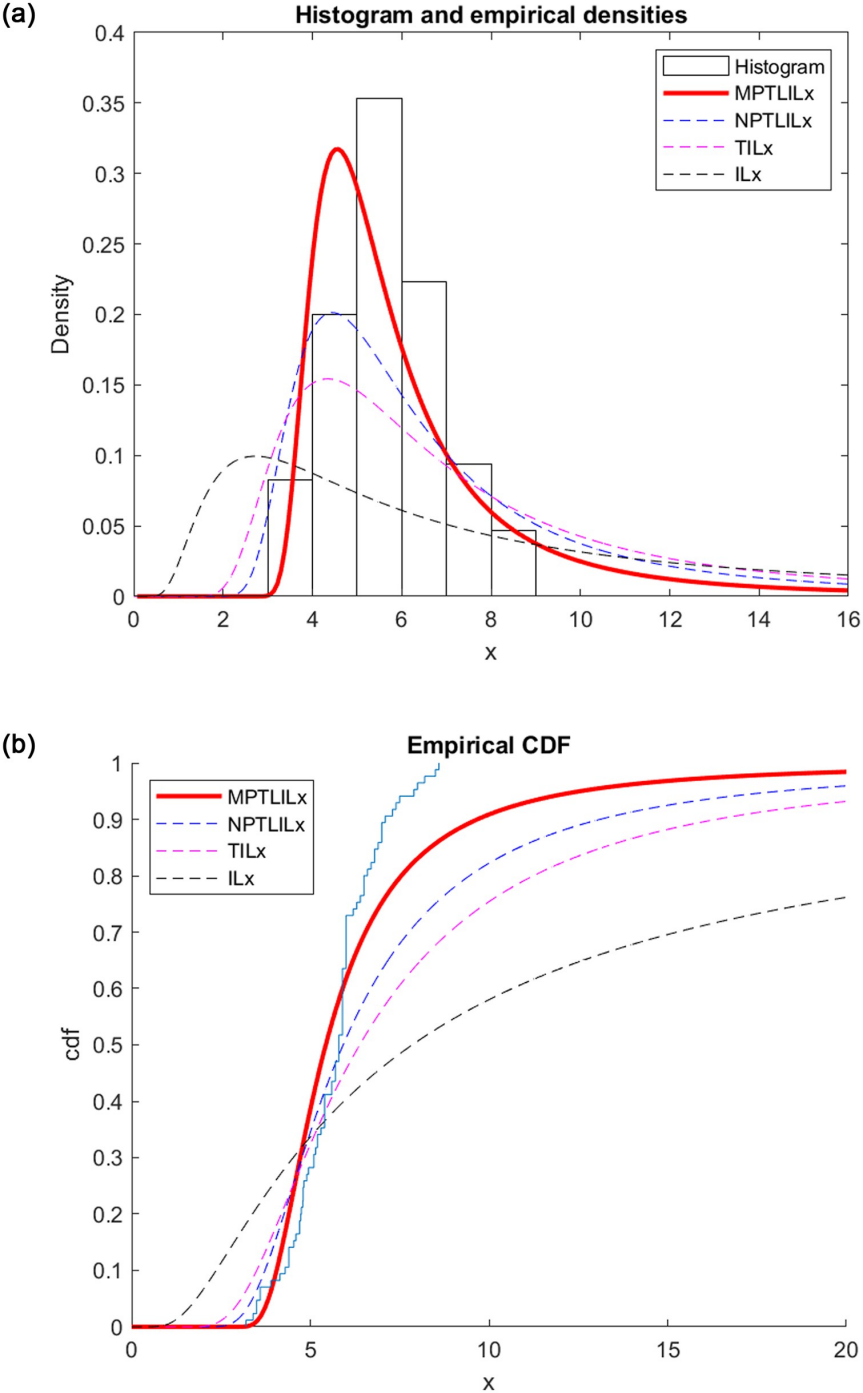
Graphical representation of the empirical pdfs and CDFs (dataset I). (a) pdfs representation. (b) CDFs representation.

The pdf and CDF for the dataset I are shown in Fig 3 of estimated fdps and fdcs for dataset I. They demonstrate that compared to the NPTLILx, TILx and ILx models, the MPTLILx model more closely describes the data.

**Fig 4:**
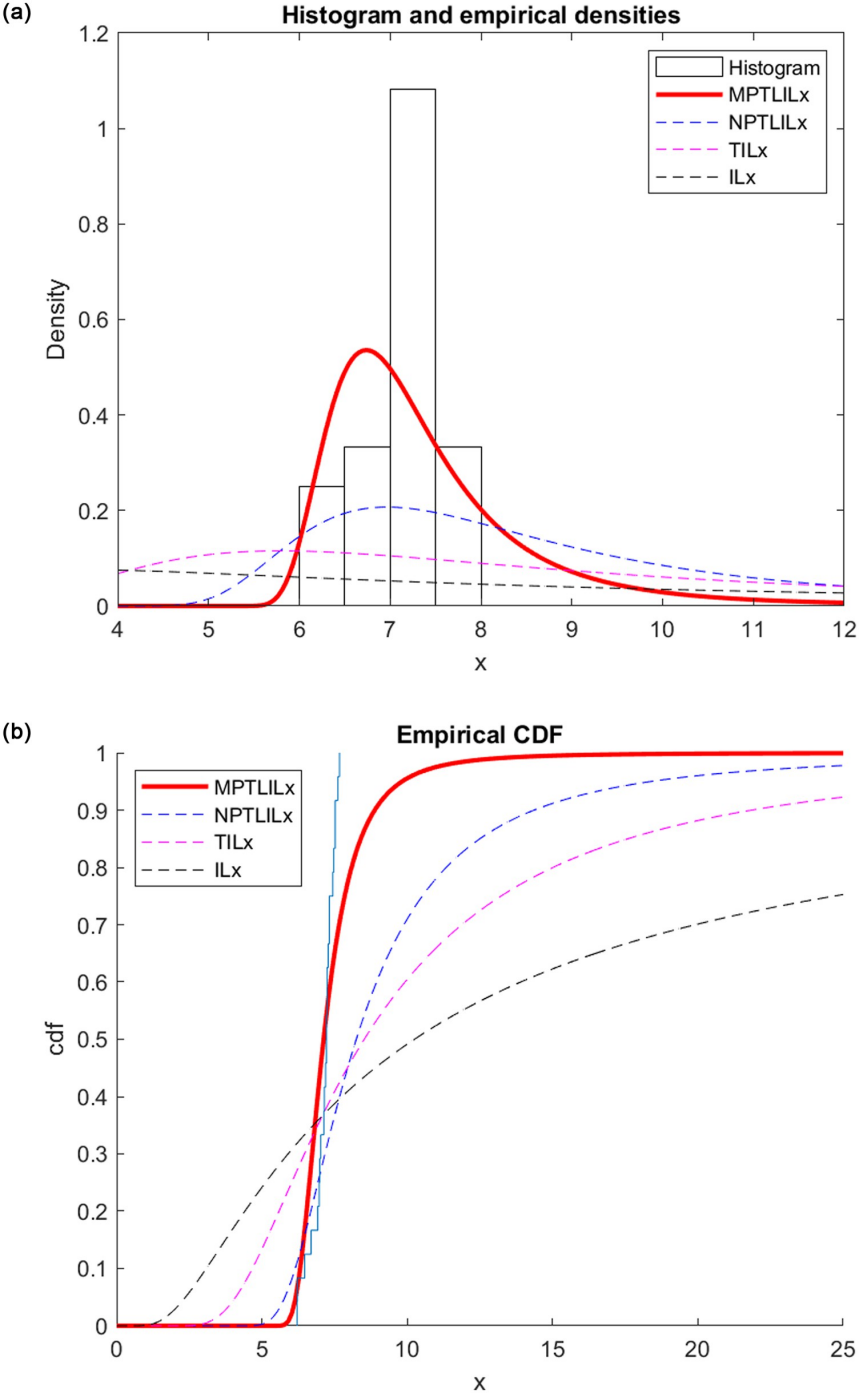
Graphical representation of the empirical pdfs and CDFs (dataset II). (a) pdfs representation. (b) CDFs representation.

For data set II the repartition functions and the pdf are shown in Fig 4 of estimated pdfs and CDFs for dataset II. They demonstrate that compared to the NPTLILx, TILx and ILx models, the MPTLILx model more effectively approches the data.

We conclude that the MPTLILx model fits datasets I and II better than the NPTLILx, TILx and ILx models based on the examination of the Tables (4 and 5) and figures (Figs 3 and 4). The financial and hydrological data can be applied to this new model due to its flexibility.

## 6. Conclusion

Within the scope of this work, we have presented and investigated a novel distribution, the so-called MPTLILx i.e. a special member of the NPTL-G family. This new model has as a basic distribution, the exponential of the modified inverse Lomax distribution. This basic distribution has been inserted in the NPTL-G family to have the MPTLILx distribution. This new distribution is a novelty in the theory of statistical distributions and can be adapted to several fields of application. We have presented various mathematical properties including Rényi Entropy, qf, series development and moment weighted probabilities. This qf increases as the three parameters increase, so we conjecture that for financial data (stock market data for example) our model would still be suitable for statistical analysis. We estimated the unknown parameters using a classical method. The MPTLILx model was then applied to illustrate the examination of two practical data sets. The MPTLILx model was shown to be better compared to its competitors in terms of AIC, CAIC, BIC, HQIC and other famous statistical metrics as shown in the application section. Further than the scope of this study, we feel that the MPTLILx model has the potential to be very helpful for a variety of applications in the real world.

Among the interesting perspectives, we could create a family of distributions by combining the NPTL-G families with another distribution in order to increase the number of parameters and to have much more flexible models than the existing ones. We recall that the best model is not always the one having more parameters. This work requires further investigation, which we leave to future works.

## 7. Future work and upcoming studies

We are planning in future to work on the T-X transformation to produce a new distribution capable of modelling new life time events. Also, we will work on bi variate distribution and we will make some studies on copula and other properties for the new distribution. At last but not least, we will apply the new distribution on some engineering and accelerated data to study the reliability function behaviour using accoutred data.
